# Multimodal evidence for the association between muscle mass and NAFLD: insights from hospital-based data, NHANES and mendelian randomization

**DOI:** 10.3389/fgene.2026.1821445

**Published:** 2026-05-26

**Authors:** Weidong Xie, Sen Li, Shaoliang Han

**Affiliations:** 1 Department of Head and Neck Surgery, Affiliated Hospital of Jiaxing University (The First Hospital of Jiaxing), Jiaxing, China; 2 School of Basic Medical Sciences, Wenzhou Medical University, Wenzhou, China; 3 Department of Gastrointestinal Surgery, The First Affiliated Hospital of Wenzhou Medical University, Wenzhou, China

**Keywords:** mendelian randomization, NHANES, non-alcoholic fatty liver disease, sarcopenia, skeletal muscle mass

## Abstract

**Background:**

Sarcopenia has been suggested as a potential risk factor for non-alcoholic fatty liver disease (NAFLD), but the causal relationship remains unclear.

**Methods:**

We conducted a multimodal study combining hospital-based data, population survey, and Mendelian randomization (MR). A total of 2,384 patients with acute appendicitis (2016–2025) were analyzed using appendicular skeletal muscle mass index (ASMI) as a sarcopenia marker and CT-defined NAFLD as outcome. Logistic regression and SIMEX correction were applied. The association was validated in 21,696 NHANES participants (1999–2018) using the United States Fatty Liver Index (USFLI) and double-SIMEX correction. Finally, two-sample and multivariable MR were performed using GWAS summary data.

**Results:**

Higher muscle mass was consistently associated with a lower NAFLD risk in hospital-based (OR = 0.16, 95% CI 0.03–0.82, P = 0.03) and NHANES populations (OR = 0.02, 95% CI 0.01–0.04, P < 0.001). MR analyses suggested a potential causal inverse relationship (OR = 0.91, 95% CI 0.84–0.99, P = 0.01), independent of BMI, cholesterol, and triglycerides (OR = 0.82, 95% CI 0.72–0.94, P = 0.003).

**Conclusion:**

This multimodal evidence suggested low muscle mass as a causal risk factor for NAFLD. Interventions that maintain or increase muscle mass may reduce NAFLD risk.

## 1 Introduction

Non-alcoholic fatty liver disease (NAFLD) is the most prevalent chronic liver disease worldwide, affecting approximately 25% of the adult population (Younossi et al., 2016a). NAFLD can further progress to liver cirrhosis and hepatocellular carcinoma, resulting in significant clinical and economic burdens (Nguyen et al., 2019; Younossi et al., 2016b). Moreover, NAFLD is associated with various metabolic comorbidities, including obesity, type 2 diabetes mellitus, dyslipidemia, and hypertension, all of which can exacerbate disease progression and adverse outcomes (Zou et al., 2020). Despite extensive research, the pathophysiological mechanisms underlying NAFLD are still not fully understood, highlighting the need for further investigation into potential risk factors and intervention strategies.

Sarcopenia, characterized by the progressive loss of skeletal muscle mass, strength, and function (Cruz-Jentoft et al., 2019), is increasingly recognized as an important risk factor for NAFLD. Up to 60% of patients with advanced liver disease exhibit sarcopenia, which is associated with poor prognosis (Bhanji et al., 2017). Although certain common pathogenic mechanisms, such as insulin resistance (Postic and Girard, 2008) and chronic inflammation (Hong et al., 2014; Kawaguchi et al., 2022), have been proposed to explain the association between sarcopenia and NAFLD, the causal relationship remains uncertain (Joo and Kim, 2023), and studies have yielded inconsistent results even within the same ethnic populations (Hong et al., 2014; Zhai et al., 2018).

Therefore, in this study, we aimed to explore the relationship between muscle mass (one of the diagnostic criteria for sarcopenia) and NAFLD. Initially, we conducted a preliminary investigation using hospital-based data, followed by validation using the National Health and Nutrition Examination Survey (NHANES) dataset. Finally, Mendelian randomization (MR) analysis was performed to evaluate the causal relationship between muscle mass and NAFLD at the genetic level.

## 2 Methods

### 2.1 Hospital-based exploration

#### 2.1.1 Data source and population

Due to local policy constraints, it was challenging to obtain data from the general population (defined here as individuals who are generally healthy). Therefore, we initially explored the relationship between muscle mass and NAFLD using data from patients admitted to the Gastrointestinal Surgery Department of the First Affiliated Hospital of Wenzhou Medical University from 2016 to 2025 with a diagnosis of acute appendicitis. The diagnosis of acute appendicitis was confirmed by postoperative pathology.

To ensure the reliability of our analysis, we applied the following exclusion criteria: (1) incomplete data, such as missing height, weight, or abdominal computed tomography (CT) scans; (2) severe wasting conditions or chronic catabolic states, such as malignant tumors, organ failure (e.g., heart failure), tuberculosis, bronchiectasis, autoimmune diseases and other systemic inflammation, endocrine and metabolic disorders (e.g., hyperthyroidism), chronic digestive disorders (e.g., chronic intestinal obstruction), other chronic catabolic states (e.g., severe chronic anemia), or ongoing chemotherapy; and (3) patients diagnosed with hepatitis B or C.

This study was approved by the Ethics Committee of the First Affiliated Hospital of Wenzhou Medical University (KY2025-R176).

#### 2.1.2 Measurement of muscle mass and diagnosis of NAFLD

In this study, appendicular skeletal muscle mass (ASM) was used as an indicator of sarcopenia. ASM was calculated using an estimation formula developed by Wen et al., specifically validated for the Chinese population, showing good agreement with dual-energy X-ray absorptiometry (DXA) measurements (Wen et al., 2011; Zhao et al., 2024). The specific calculation formula is as follows:

$$ASM=0.193\times \text{weight }\left(\text{kg}\right)+0.107\times \text{height }\left(\text{cm}\right)$$

$$-4.157\times \text{sex }\left(\text{male}=1,\text{female}=2\right)-0.037\times \text{age }\left(\text{year}\right)-2.631$$

Appendicular skeletal muscle mass index (ASMI) was obtained by standardizing ASM to body mass index (BMI), calculated as:

$$ASMI=\frac{\text{ASM}}{\text{BMI}}$$

Computed tomography (CT) was used to diagnose NAFLD in patients (Yes or No).

#### 2.1.3 Covariates

Covariates included sex, age, BMI, smoking history, alcohol consumption history, diabetes mellitus history, and hypertension history. BMI was categorized into three groups: underweight (<18.5), normal weight (18.5–25), and overweight (>25). Smoking and alcohol consumption histories were obtained from patient self-reports. Histories of diabetes mellitus and hypertension were collected based on patient self-report or current use of relevant medications. The population included in this study was of East Asian ethnicity.

#### 2.1.4 Statistical analysis

Continuous variables were expressed as mean (standard deviation, SD), and categorical variables were presented as percentages. Continuous variables were compared using Student’s t-test if normally distributed; otherwise, the Wilcoxon rank-sum test was used. The chi-square test was employed for comparisons between categorical variables. Restricted cubic splines (RCS) were applied to evaluate potential nonlinear relationships between muscle mass and NAFLD. Logistic regression models were used to analyze the association between muscle mass and NAFLD. Model 1 was unadjusted. Model 2 was adjusted for sex, age, and BMI. Model 3 was further adjusted for smoking history, alcohol consumption history, hypertension history, and diabetes mellitus history. Given that ASM was estimated using a formula, measurement errors were possible. Thus, the SIMEX method (Cook and Stefanski, 2012) was employed to correct potential measurement errors and assess the robustness of the results. Jackknife variance estimation was used in the SIMEX method. All analyses were performed using R software (version 4.3.3).

### 2.2 NHANES

#### 2.2.1 Data source and population

This study utilized data from the NHANES conducted between 1999 and 2018. All data are publicly available on the NHANES website (https://www.cdc.gov/nchs/nhanes). The exclusion criteria were as follows: (1) incomplete data, including missing variables such as smoking history, alcohol consumption history, and variables required to calculate the United States Fatty Liver Index (USFLI) and ASM; (2) excessive alcohol consumption (defined as >21 drinks per week for men and >14 drinks per week for women) (Chalasani et al., 2012); and (3) missing viral hepatitis data or presence of viral hepatitis (positive serum hepatitis B surface antigen or hepatitis C antibody). The NHANES protocol was approved by the Ethics Review Board of the National Center for Health Statistics (NCHS) (https://www.cdc.gov/nchs/nhanes/irba98.htm), and all participants provided written informed consent before enrollment.

#### 2.2.2 Measurement of muscle mass and diagnosis of NAFLD

In this study, ASM was used as an indicator of muscle mass. ASM was estimated using a formula developed by Shi et al. for the NHANES population, which has demonstrated good reliability (Shi et al., 2023). The formula is as follows:

$$ASM=0.485\times 0.998^{age\left(year\right)}\times 0.814^{female}\times 1.006^{height\left(cm\right)}\times {weight\left(kg\right)}^{0.680}$$

ASMI was derived by standardizing ASM to BMI, calculated as:

$$ASMI=\frac{\text{ASM}}{\text{BMI}}$$

NAFLD was diagnosed using the USFLI, a non-invasive method for assessing NAFLD with a score ranging from 0 to 100, which has been widely applied in NAFLD-related research (Younossi et al., 2020; Mazidi et al., 2020; Ruan et al., 2022). In this study, NAFLD was defined as USFLI ≥ 30 (Ruhl and Everhart, 2015).

#### 2.2.3 Covariates

Covariates included sex, age, race, educational level, BMI, smoking status, and alcohol consumption. BMI was categorized according to the criteria described previously. Smoking status was classified as never smokers (smoked fewer than 100 cigarettes in their lifetime), former smokers (smoked more than 100 cigarettes in their lifetime but had quit), and current smokers (smoked more than 100 cigarettes in their lifetime and were still smoking). Alcohol consumption was categorized as never, 1–5 drinks per month, 5–10 drinks per month, and more than 10 drinks per month. Diabetes mellitus was defined as glycated hemoglobin (HbA1c) > 6.5%, fasting blood glucose > 126 mg/dL, or current use of glucose-lowering medications (including insulin). Hypertension was defined as systolic blood pressure > 140 mmHg, diastolic blood pressure > 90 mmHg, or current use of antihypertensive medications.

#### 2.2.4 Statistical analysis

In accordance with the NHANES analytic guidelines, complex survey design and sampling weights were incorporated into all analyses (Johnson et al., 2013). Descriptive statistics were conducted as described previously. Weighted restricted cubic splines were applied to assess potential nonlinear relationships between muscle mass and NAFLD. Weighted logistic regression models were used to evaluate the association between muscle mass and NAFLD. Model 1 was unadjusted. Model 2 was adjusted for sex, age, BMI, race, and educational level. Model 3 was further adjusted for smoking history, alcohol consumption, hypertension history, and diabetes mellitus history. Because ASM was estimated using a formula and the diagnosis of NAFLD was also formula-based, this may introduce dual measurement error. Therefore, the double-SIMEX method (Holcomb, 1999; Guolo, 2017) was applied to correct potential errors and to assess the robustness of the results. All analyses were performed using R software (version 4.3.3).

### 2.3 Mendelian randomization

#### 2.3.1 GWAS data source for muscle mass

As there are currently no genome-wide association study (GWAS) data directly reporting ASM, and because ASM is nearly equivalent to appendicular lean mass (ALM), we used ALM as a proxy for ASM in this analysis. ALM data were obtained from a UK Biobank–based study conducted by Pei et al., in which ALM was measured using bioelectrical impedance analysis, this study included 450,243 participants (Pei et al., 2020).

#### 2.3.2 GWAS data source for NAFLD

We used the GWAS summary statistics reported by Ghodsian et al., which were derived from a meta-analysis of four cohort studies and included 8,434 cases and 770,180 controls (Ghodsian et al., 2021).

#### 2.3.3 GWAS data sources for other variables

GWAS data for BMI were obtained from the Genetic Investigation of Anthropometric Traits (GIANT) Consortium, which included 322,154 individuals of European ancestry (Locke et al., 2015). GWAS summary statistics for total cholesterol and triglycerides were obtained from the study by J Kettunen J. et al., which included 21,491 individuals of European ancestry (Kettunen et al., 2016).

#### 2.3.4 Selection of genetic instrumental variables (IVs)

To ensure the validity of the instrumental variables (IVs), we first applied a genome-wide significance threshold of P < 5 × 10^−8^ for SNP selection. We then excluded SNPs in linkage disequilibrium (LD) using the criteria r^2^ < 0.001 with a clumping window of 10,000 kb. For SNPs not directly available in the outcome dataset, proxy SNPs were allowed if the LD r^2^ > 0.8. Additionally, we calculated the F-statistic for each IV (Palmer et al., 2012; Levin et al., 2020; Gill et al., 2019) using the following formula, and an F value greater than 10 was required to minimize potential bias from weak instruments (Burgess et al., 2011).

$$R^{2}=\frac{2\times \beta^{2}\times EAF\times \left(1-EAF\right)}{2\times \beta^{2}\times \left(1-EAF\right)+2\times {SE}^{2}\times N\times EAF\times \left(1-EAF\right)}$$

$$F=\frac{R^{2}}{1-R^{2}}\times \frac{N-K-1}{K}$$

#### 2.3.5 Mendelian randomization analysis

In this study, the inverse variance weighted (IVW) method was used as the primary approach, with weighted median (Bowden et al., 2016), MR-Egger (Bowden et al., 2015), and MR-PRESSO (Verbanck et al., 2018) serving as complementary analyses. Cochran’s Q test was applied to assess heterogeneity (Greco et al., 2015), and the MR-Egger intercept test was used to evaluate horizontal pleiotropy (Bowden et al., 2015). The leave-one-out method was used to evaluate whether the MR results were excessively influenced by any single SNP. Additionally, because a certain degree of sample overlap existed between the exposure and outcome datasets, we used MRlap method to evaluate whether sample overlap might excessively bias the results. If the IVW estimates before and after MRlap correction showed no significant difference, the uncorrected IVW results were reported; otherwise, the corrected IVW results were used (Mounier and Kutalik, 2023). For MRlap, a more stringent threshold (P < 5 × 10^−10^) was applied for selecting instrumental variables. Detailed information on MRlap is available at https://github.com/n-mounier/MRlap. Multivariable Mendelian Randomization (MVMR) was performed to assess the independent effect of muscle mass on NAFLD after adjusting for BMI, total cholesterol and triglycerides. Reverse Mendelian randomization was also be performed.

## 3 Results

### 3.1 Analysis based on hospital data

#### 3.1.1 Demographic characteristics

After strict screening, a total of 2,384 eligible patients with postoperative pathological diagnoses of acute appendicitis were included, among whom 410 had CT-reported NAFLD. Detailed demographic characteristics are presented in Table 1.

**TABLE 1:** Demographic characteristics of the hospital data.

Characteristic	Overall N = 2,384^a^	No NAFLD N = 1,974^a^	NAFLD N = 410^a^	P-value^b^
Age	43 (17)	42 (17)	45 (16)	0.011
Sex	​	​	​	<0.001
Male	1,263 (53%)	980 (50%)	283 (69%)	​
Female	1,121 (47%)	994 (50%)	127 (31%)	​
BMI	​	​	​	<0.001
<18.5	201 (8.4%)	196 (9.9%)	5 (1.2%)	​
18.5–25	1,576 (66%)	1,398 (71%)	178 (43%)	​
>25	607 (25%)	380 (19%)	227 (55%)	​
Smoke	​	​	​	0.021
Yes	333 (14%)	261 (13%)	72 (18%)	​
No	2,051 (86%)	1,713 (87%)	338 (82%)	​
Alcohol	​	​	​	>0.9
Yes	264 (11%)	218 (11%)	46 (11%)	​
No	2,120 (89%)	1,756 (89%)	364 (89%)	​
Hypertension	​	​	​	<0.001
Yes	226 (9.5%)	164 (8.3%)	62 (15%)	​
No	2,158 (91%)	1,810 (92%)	348 (85%)	​
Diabetes	​	​	​	<0.001
Yes	94 (3.9%)	63 (3.2%)	31 (7.6%)	​
No	2,290 (96%)	1,911 (97%)	379 (92%)	​
ASMI	0.85 (0.18)	0.85 (0.18)	0.86 (0.17)	0.13

^a^ Mean (SD); n (%).

^b^ Wilcoxon rank sum test; Pearson’s Chi-squared test; Student’s t-test.

#### 3.1.2 RCS analysis

**FIGURE 1:**
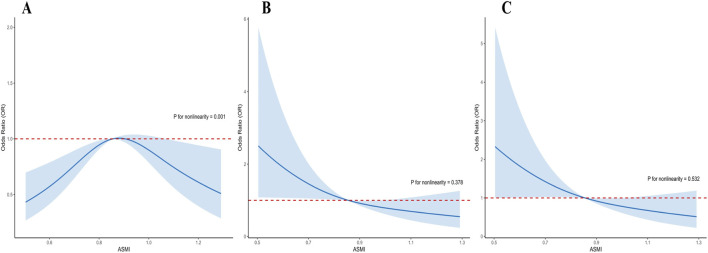
RCS curves (Hospital Data). Across progressively adjusted models, higher ASMI was associated with a lower probability of NAFLD. **(A)** Model 1, Unadjusted. **(B)** Model 2, Adjusted for age, sex, and BMI. **(C)** Model 3, Further adjusted for smoking history, alcohol consumption, hypertension history, and diabetes mellitus history.

In Model 1, without adjustment for any covariates, a nonlinear relationship was observed between muscle mass and NAFLD (P = 0.001). After further adjustment for sex, age, and BMI in Model 2, the nonlinear association disappeared, and a linear relationship emerged (P = 0.378). Finally, after additional adjustment for smoking history, alcohol consumption, hypertension history, and diabetes mellitus history, the linear relationship persisted (P = 0.532), with the risk of NAFLD progressively decreasing as muscle mass increased. Details are shown in Figure 1.

#### 3.1.3 Logistic regression

In Model 1, without adjustment for any covariates, muscle mass was not significantly associated with NAFLD (OR = 1.44, 95% CI: 0.79–2.60, P = 0.20). After adjusting for sex, age, and BMI in Model 2, higher muscle mass was significantly associated with a reduced risk of NAFLD (OR = 0.16, 95% CI: 0.03–0.83, P = 0.03). In Model 3, with additional adjustment for smoking history, alcohol consumption, hypertension history, and diabetes mellitus history, higher muscle mass remained protective against NAFLD (OR = 0.16, 95% CI: 0.03–0.82, P = 0.03). After correcting for potential bias using the SIMEX method, the results remained robust, consistently indicating an inverse association between muscle mass and the risk of NAFLD (Table 2).

**TABLE 2:** Logistic regression and SIMEX correction results (Hospital Data).

ASMI increment	​	Logistic			SIMEX		
	Model	OR	95% CI	P value	OR	95% CI	P value
Per ASMI	Model1	1.44	0.79, 2.60	0.2	1.51	0.78, 2.91	0.2
	Model2	0.16	0.03, 0.83	0.03	0.05	0.005, 0.59	0.02
	Model3	0.16	0.03, 0.82	0.03	0.05	0.005, 0.58	0.02
Per SD	Model1	1.07	0.96, 1.19	0.2	1.08	0.96, 1.21	0.2
	Model2	0.72	0.54, 0.97	0.03	0.59	0.39, 0.91	0.02
	Model3	0.72	0.53, 0.97	0.03	0.59	0.39, 0.91	0.02

Model1: Unadjusted.

Model2: Ajusted for age, sex, and BMI.

Model3: Further adjusted for smoking history, alcohol consumption, hypertension history, and diabetes mellitus history.

### 3.2 NHANES

#### 3.2.1 Demographic characteristics

After applying the selection criteria, a total of 21,696 participants were included, among whom 7,468 were diagnosed NAFLD. Detailed demographic characteristics are presented in Table 3.

**TABLE 3:** Demographic characteristics of the NHANES population.

Characteristic	Overall N = 21,696^a^	No NAFLD N = 14,228^a^	NAFLD N = 7468^a^	P-value^b^
Age	47 (17)	45 (17)	51 (16)	<0.001
Sex	​	​	​	<0.001
Male	10,494 (48%)	6,318 (44%)	4,176 (58%)	​
Female	11,202 (52%)	7,910 (56%)	3,292 (42%)	​
BMI	​	​	​	<0.001
<18.5	347 (1.6%)	338 (2.3%)	9 (<0.1%)	​
18.5–25	6,156 (29%)	5,755 (41%)	401 (3.7%)	​
>25	15,193 (69%)	8,135 (57%)	7,058 (96%)	​
Race	​	​	​	<0.001
Non-hispanic white	9,799 (68%)	6,448 (68%)	3,351 (70%)	​
Non-hispanic black	4,189 (11%)	3,328 (13%)	861 (6.2%)	​
Mexican american	3,923 (8.5%)	1,883 (6.3%)	2,040 (13%)	​
Other hispanic	1,852 (5.4%)	1,143 (5.3%)	709 (5.6%)	​
Other	1,933 (7.2%)	1,426 (7.9%)	507 (5.6%)	​
Education	​	​	​	<0.001
Less than 9th grade	2,621 (5.7%)	1,346 (4.7%)	1,275 (7.9%)	​
9–11th grade	3,216 (11%)	1,975 (9.8%)	1,241 (13%)	​
High school Grad/GED	4,972 (24%)	3,250 (23%)	1,722 (25%)	​
Some college or AA degree	6,117 (31%)	4,095 (30%)	2,022 (33%)	​
College graduate or above	4,770 (29%)	3,562 (32%)	1,208 (22%)	​
Smoke	​	​	​	<0.001
Current smoker	4,422 (20%)	3,066 (21%)	1,356 (18%)	​
Former smoker	5,531 (25%)	3,160 (22%)	2,371 (32%)	​
Never smoker	11,743 (55%)	8,002 (56%)	3,741 (51%)	​
Alcohol	​	​	​	<0.001
Non-drinker	5,929 (23%)	3,809 (22%)	2,120 (25%)	​
1–5 drinks/month	11,439 (52%)	7,363 (51%)	4,076 (54%)	​
5–10 drinks/month	1,511 (8.9%)	1,090 (9.5%)	421 (7.6%)	​
10+ drinks/month	2,817 (16%)	1,966 (17%)	851 (13%)	​
Hypertension	​	​	​	<0.001
Yes	8,238 (33%)	4,263 (24%)	3,975 (51%)	​
No	13,458 (67%)	9,965 (76%)	3,493 (49%)	​
Diabetes	​	​	​	<0.001
Yes	3,577 (12%)	1,128 (5.6%)	2,449 (27%)	​
No	18,119 (88%)	13,100 (94%)	5,019 (73%)	​
ASMI	0.79 (0.19)	0.80 (0.19)	0.76 (0.18)	<0.001

^a^ n (unweighted) (%); % (weighted), Mean (SD).

^b^ chi-squared test with Rao & Scott’s second-order correction; Wilcoxon rank-sum test for complex survey samples.

#### 3.2.2 Weighted RCS analysis

**FIGURE 2:**
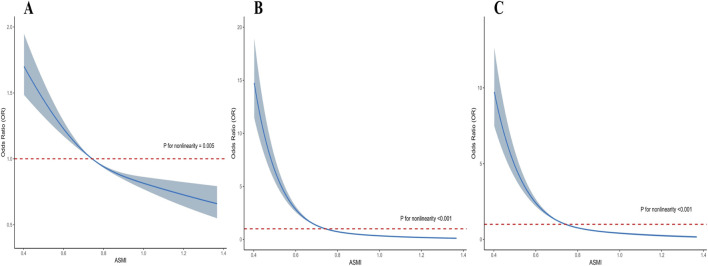
Weighted RCS Curve (NHANES). Higher ASMI consistently correlated with reduced NAFLD risk across all adjustment models, demonstrating a robust inverse relationship. **(A)** Model 1, Unadjusted. **(B)** Model 2, Adjusted for age, sex, BMI, race, and educational level. **(C)** Model 3, Further adjusted for smoking history, alcohol consumption, hypertension history, and diabetes mellitus history.

In Model 1, without any covariate adjustment, a nonlinear relationship was observed between muscle mass and the risk of NAFLD (P = 0.005), with the risk of NAFLD steadily decreasing as muscle mass increased. After adjusting for sex, age, BMI, race, and educational level, the nonlinear association persisted (P < 0.001), and muscle mass remained inversely associated with NAFLD risk. Further adjustment for smoking history, alcohol consumption, hypertension history, and diabetes mellitus history did not change this pattern (P < 0.001), and higher muscle mass continued to demonstrate a protective effect against NAFLD. Detailed results are shown in Figure 2.

#### 3.2.3 Weighted logistic regression

In Model 1, without adjustment for any covariates, appendicular skeletal muscle mass was inversely associated with the risk of NAFLD (OR = 0.36, 95% CI: 0.29–0.45, P < 0.001). After further adjustment for sex, age, BMI, race, and educational level, the association remained statistically significant (OR = 0.01, 95% CI: 0.004–0.02, P < 0.001). Finally, after adjusting for the remaining covariates, appendicular skeletal muscle mass continued to demonstrate a robust protective effect against NAFLD (OR = 0.02, 95% CI: 0.01–0.04, P < 0.001). After correcting for potential bias using the double-SIMEX method, the results remained robust, further supporting that higher skeletal muscle mass is associated with a reduced risk of NAFLD. Detailed results are presented in Table 4.

**TABLE 4:** Weighted logistic regression and Double-SIMEX correction results (NHANES).

ASMI increment	​	Logistic			Double-SIMEX		
	Model	OR	95% CI	P value	OR	95% CI	P value
Per ASMI	Model1	0.36	0.29, 0.45	<0.001	0.23	0.06, 0.996	0.049
	Model2	0.01	0.004, 0.02	<0.001	0.004	<0.001, 0.37	0.02
	Model3	0.02	0.01, 0.04	<0.001	0.01	<0.001, 0.40	0.01
Per SD	Model1	0.82	0.79, 0.86	<0.001	0.76	0.59, 1.00	0.049
	Model2	0.40	0.35, 0.45	<0.001	0.35	0.17, 0.83	0.02
	Model3	0.47	0.42, 0.54	<0.001	0.44	0.23, 0.84	0.01

Model 1: Unadjusted.

Model 2: Adjusted for age, sex, BMI, race, and educational level.

Model 3: Further adjusted for smoking history, alcohol consumption, hypertension history, and diabetes mellitus history.

### 3.3 Mendelian randomization

#### 3.3.1 Two-sample mendelian randomization

After selecting IVs according to the aforementioned criteria, no weak instruments were identified (Supplementary Material 2). We subsequently performed a two-sample Mendelian randomization analysis, which revealed an inverse association between muscle mass and NAFLD (OR = 0.91, 95% CI: 0.84–0.99, P = 0.01). The MR-PRESSO results (OR = 0.92, 95% CI: 0.85–0.997, P = 0.04) were consistent with the IVW estimates. The weighted median (OR = 0.97, P = 0.64) and MR-Egger (OR = 0.97, P = 0.73) estimates were directionally consistent with the IVW result but did not reach statistical significance. Detailed results are presented in Table 5. Sensitivity analyses found no evidence of horizontal pleiotropy (Supplementary Material Table 1). Reverse Mendelian randomization yielded no statistically significant results (Supplementary Material Table 2). Collectively, these findings suggest a potential causal relationship between higher appendicular muscle mass and a lower risk of NAFLD. Visualized MR results are provided in the Supplementary Material 2 Figures 1-4.

**TABLE 5:** Two-sample Mendelian randomization results.

Exposure	Method	OR^a^	95% CI^a^	p-value
ALM	MR egger	0.97	0.80, 1.16	0.73
	Weighted median	0.97	0.86, 1.09	0.64
	IVW	0.91	0.84, 0.99	0.01
	MR-PRESSO	0.92	0.85, 0.997	0.04

^a^ OR, odds ratio; CI, Confidence Interval.

We further performed MRlap analysis to correct for potential bias introduced by sample overlap between the exposure and outcome populations. MRlap results consistently demonstrated an inverse causal relationship between appendicular muscle mass and NAFLD risk (Supplementary Material Table 3).

#### 3.3.2 Multivariable Mendelian Randomization

In the multivariable Mendelian randomization analysis, we adjusted for BMI, total cholesterol, and triglycerides. The results (Supplementary Material Table 4) showed that, even after adjusting for these variables, skeletal muscle mass remained significantly protective against the risk of NAFLD (OR = 0.82, 95% CI: 0.72–0.94, P = 0.003). This suggests that higher appendicular skeletal muscle mass may serve as an independent protective factor against NAFLD, with evidence supporting a potential causal relationship.

## 4 Discussion

In this study, we comprehensively evaluated the relationship between muscle mass and NAFLD from multiple perspectives. Our findings consistently showed that, whether based on real-world hospital data, large-scale survey data, or genetic evidence, an increase in appendicular muscle mass was associated with a progressively lower risk of NAFLD. Furthermore, the Mendelian randomization analysis provided evidence supporting a potential causal relationship between higher appendicular muscle mass and reduced NAFLD risk.

Regarding muscle mass, most previous studies have reported that sarcopenia is a risk factor for NAFLD (Hong et al., 2014; Kim et al., 2016; Lee et al., 2015; Lee et al., 2016), which is consistent with our findings. Skeletal muscle is now recognized as an endocrine organ (Febbraio and Pedersen, 2005), capable of secreting myokines that participate in metabolic processes within the muscle itself, as well as in the liver and adipose tissue (Carson, 2017; Oh and Lee, 2020). For example, irisin, a myokine involved in the browning of white adipose tissue and thermogenesis (Boström et al., 2012), has been shown to be independently and inversely associated with hepatic fat content (Zhang et al., 2013). Additionally, myostatin (McPherron et al., 1997), a skeletal muscle–derived inhibitor of myogenesis, is significantly upregulated during the progression of sarcopenia (Kumagai et al., 2021). In animal models, exposure to myostatin has been shown to promote hepatic stellate cells to increase the expression of pro-fibrotic proteins (Delogu et al., 2019). These findings provide biological insights and mechanistic plausibility for the association between muscle mass and NAFLD. However, the present study focused solely on muscle mass, while the effects of muscle function (e.g., grip strength and gait speed) on NAFLD remain largely unexplored. Given that muscle function may play an independent role in the pathogenesis of NAFLD, we believe this represents an important direction for future research.

According to the current “multiple hits” hypothesis (Polyzos et al., 2012), in addition to genetic factors, environmental factors play a key role in the pathogenesis of NAFLD, such as sedentary behavior and Western-style dietary patterns (Vachliotis et al., 2022). In our study, beyond demonstrating a causal association between muscle mass and NAFLD, we also observed in the NHANES population that individuals with NAFLD not only had a higher prevalence of elevated BMI but also a lower mean ASMI. A possible explanation is that factors such as physical inactivity lead to reduced muscle mass and energy expenditure, which in turn contribute to obesity (Biolo et al., 2014). Some scholars have suggested that, although muscle loss and fat accumulation are often regarded as part of the natural aging process, they are largely the consequence of physical inactivity and sedentary behavior (Benton et al., 2011). Previous studies have found that irisin expression is significantly reduced in obese individuals (Moreno-Navarrete et al., 2013). Moreover, excessive adipose tissue expansion alters the adipokine profile secreted by adipocytes, leading to insulin resistance and increased systemic inflammation (Polyzos et al., 2016), which may further drive the development of NAFLD. Elevated BMI is already widely recognized as a major risk factor for NAFLD. Importantly, our multivariable MR results showed that the direct protective effect of appendicular lean mass against NAFLD remained significant even after adjusting for BMI, total cholesterol, and triglycerides, further highlighting the critical role of skeletal muscle mass. Therefore, clinicians should consider implementing proactive interventions for patients with sarcopenia or obesity (particularly those with potential sarcopenic obesity), such as nutritional support, reduction of sedentary behavior, and the prescription of exercise programs, to increase muscle mass and reduce adipose tissue. However, given the limited causal evidence from MR, these clinical implications require further validation in prospective studies and larger genetic consortia. Additionally, the absence of East Asian GWAS data precluded MR analysis in this population, and the generalizability of our MR findings to East Asian individuals remains to be confirmed.

This study has several notable strengths. First, we explored the relationship between muscle mass and NAFLD from multiple perspectives—ranging from small to large sample sizes, from single-ethnicity to multi-ethnic populations, and from clinical to genetic viewpoints. We incorporated the SIMEX method to assess and correct for measurement error, thereby enhancing the robustness of our findings. Additionally, we employed the MRlap method to further validate the causal relationship between muscle mass and NAFLD, accounting for sample overlap.

This study has several limitations. First, although the opportunistic use of existing clinical imaging data for research purposes has been explored in prior studies (Penna et al., 2021; Pickhardt, 2022) and we applied strict selection criteria when using patients with acute appendicitis as a surrogate for the general population, the possibility of selection bias cannot be completely ruled out. Second, this study adopted the NAFLD definition rather than the more recently introduced Metabolic dysfunction-associated steatotic liver disease (MASLD) nomenclature. It has been demonstrated that the populations identified under the NAFLD and MASLD definitions are nearly identical, and that findings from prior NAFLD studies remain largely valid under the new framework (Ratziu et al., 2024; Song et al., 2024). Nevertheless, given that our hospital-based data spanned multiple years, a shift in diagnostic criteria during the study period would have introduced inconsistency; therefore, we retained the NAFLD definition for practical consistency. We acknowledge that the MASLD nomenclature is gaining rapid and widespread adoption, and future studies may benefit from adopting this updated terminology. Third, ASMI and USFLI share overlapping anthropometric components, and this partial structural dependency cannot be fully excluded as a contributor to the observed association. Therefore, the NHANES findings warrant validation using direct muscle mass assessment such as DXA. Finally, hepatic steatosis was assessed by CT scan in the hospital-based cohort but by the USFLI in NHANES. Although USFLI is a validated and widely accepted surrogate in large epidemiological studies, the inconsistency in outcome measurement across cohorts remains a limitation.

## 5 Conclusion

Our study suggests a potential causal association between muscle mass and NAFLD, which may inform future clinical practice and warrant further investigation.

## Data Availability

The original contributions presented in the study are included in the article/Supplementary Material, further inquiries can be directed to the corresponding authors.
