# Supplementary material for: Multimodal evidence for the association between muscle mass and NAFLD: insights from hospital-based data, NHANES and mendelian randomization
Source: Front Genet. 2026 May 26;17:1821445. doi: 10.3389/fgene.2026.1821445 (PMC13245940; doi:10.3389/fgene.2026.1821445)
Supplement: Supplementary file 1 [file DataSheet1.docx]

**Table 1: Heterogeneity and horizontal pleiotropy**

| **Heterogeneity** | | | |
| --- | --- | --- | --- |
| **Exposure** | **Outcome** | **Method** | **P value** |
| ALM | NALFD | MR Egger | ＜0.001 |
| ALM | NALFD | IVW | ＜0.001 |
| **Horizontal pleiotropy** | | | |
| **Exposure** | **Outcome** | **Method** | **P value** |
| ALM | NALFD | MR Egger | 0.73 |

**Table 2: Reverse two-sample Mendelian randomization results**

| **Exposure** | **Method** | **OR**^1^ | **95%CI**^1^ | **p-value** |
| --- | --- | --- | --- | --- |
| NAFLD | MR Egger | 1.01 | 0.89, 1.14 | 0.91 |
|  | Weighted median | 0.98 | 0.96, 0.99 | 0.01 |
|  | IVW | 0.95 | 0.91, 1.00 | 0.06 |
|  | MR-PRESSO | - | - | - |
| ^1^OR = Odds Ratio, CI = Confidence Interval, | | | |  |

Outlier-corrected MR-PRESSO results were not available because the model could not be estimated after removal of outlier SNPs due to an insufficient number of instrumental variables.

**Table 3: MRlap results**

| **Exposure** | **Outcome** | **Observed-OR** | **95%CI** | **P value** | **Corrected-OR** | **95%CI** | **P value** | **P for difference** |
| --- | --- | --- | --- | --- | --- | --- | --- | --- |
| ALM | NAFLD | 0.994 | 0.988, 1 | 0.04 | 0.994 | 0.988, 1 | 0.06 | 0.9 |

Observed-OR: Causal effect estimate observed by IVW-MR

Corrected-OR: Bias-corrected causal effect estimate

P for difference: P value corresponding to the statistical test for the difference between the observed and corrected estimates

**Table 4. Multivariable Mendelian randomization (MR) results**

| **Exposure** | **Outcome** | **OR** | **95%CI** | **P value** |
| --- | --- | --- | --- | --- |
| ALM | NAFLD | 0.82 | 0.72, 0.94 | 0.003 |
| BMI | NAFLD | 1.3 | 0.95, 1.69 | 0.10 |
| Total cholesterol | NAFLD | 0.96 | 0.85, 1.09 | 0.56 |
| Total triglycerides | NAFLD | 1.26 | 1.07, 1.50 | 0.006 |


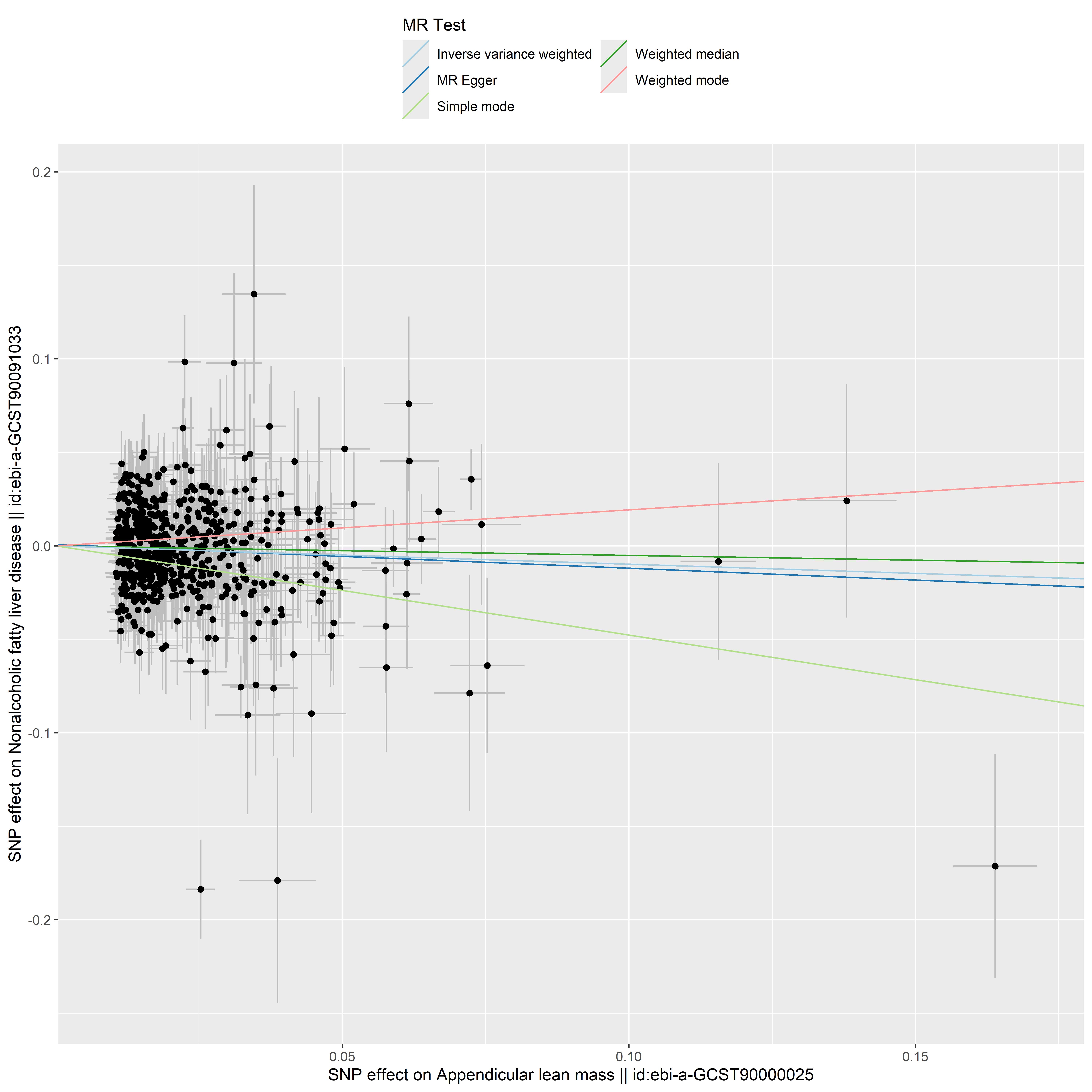


**Figure 1. Scatter plot of the association between ALM and NAFLD**

The plot shows genetic associations of instrumental SNPs with ALM (x-axis) and NAFLD (y-axis), with regression lines representing different Mendelian randomization methods.

**Figure 2. Leave-one-out analysis results**

Each analysis was repeated by sequentially removing one instrumental SNP at a time to assess whether the overall Mendelian randomization estimate was disproportionately driven by any single variant.

**

**

**Figure 3. Forest plot of the association between ALM and NAFLD**

Individual SNP-specific Wald ratio estimates and the overall pooled causal estimate are displayed to illustrate the direction and consistency of the ALM–NAFLD association.

**
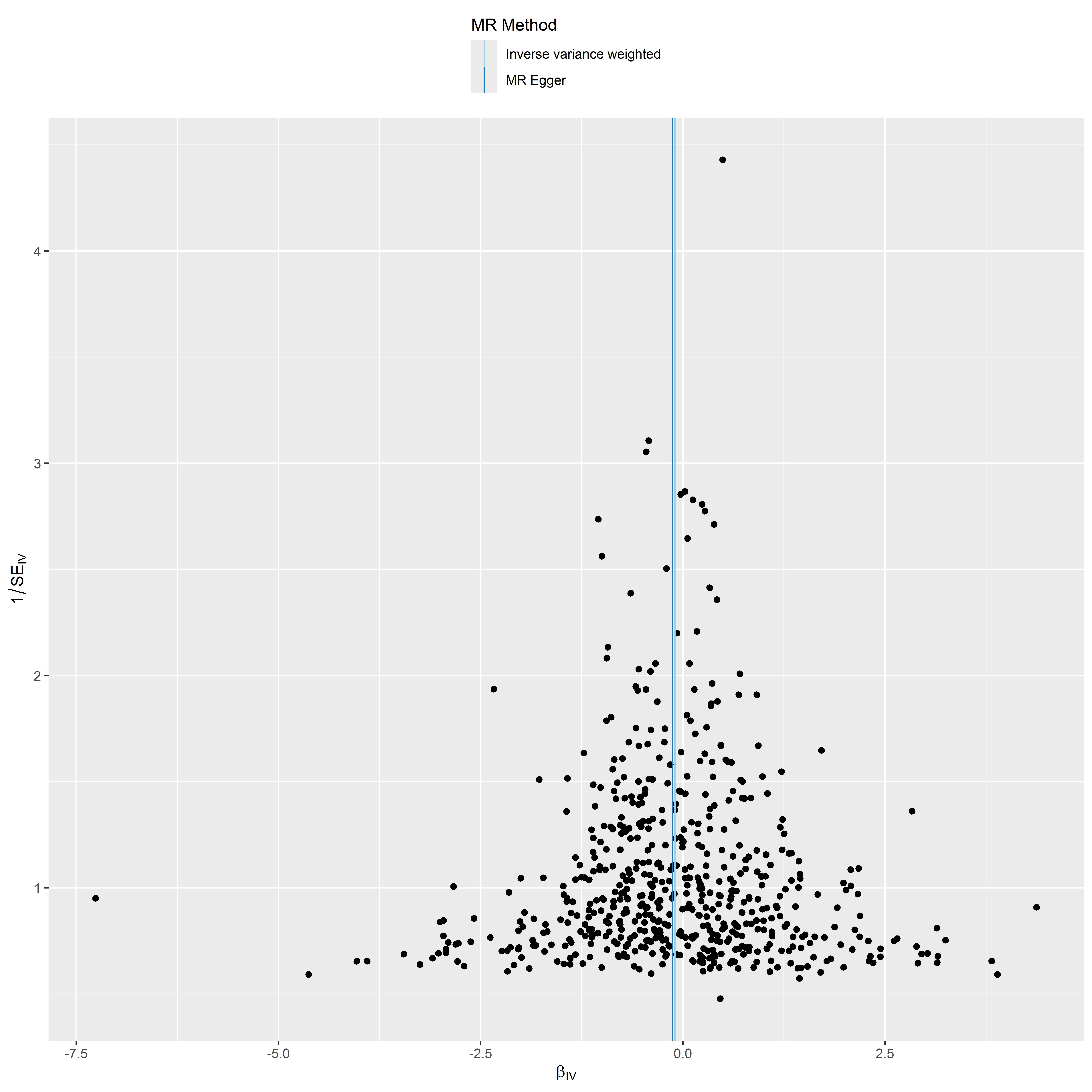
**

**Figure 4. Volcano plot of the association between ALM and NAFLD**

Each point represents an instrumental SNP, plotted by its Wald ratio estimate and statistical significance, illustrating the overall distribution and directional consistency of SNP-level effects.
